# Ocular metastases from HER2 positive breast carcinoma and the response to combination therapy with Paclitaxel and Trastuzumab: a case report

**DOI:** 10.1186/1757-1626-2-9143

**Published:** 2009-12-04

**Authors:** Konstantinos I Papageorgiou, Ajay Sinha, Alexander S Ioannidis, Neville G Davidson

**Affiliations:** 1 Department of Ophthalmology, Broomfield Hospital, Mid Essex NHS Trust, Court Road, Chelmsford, Essex, CM1 7ET, UK; 2 Capio Springfield Hospital, Lawn Lane, Springfield, Chelmsford, CM1 7GU, Essex, UK

## Abstract

**Purpose:**

Breast cancer is the most common tumour to metastasize to the uveal tract. The mean survival period after diagnosis of metastasis to the eye, ranges from 10 to 32 months. However, recent advances in therapy including the use of monoclonal antibody therapy, will hopefully improve treatment outcomes and prolong survival rates.

**Methods:**

We report a case of a 45 year old woman with a HER2 positive breast cancer, who developed two metastatic lesions in the left choroid, and the left optic nerve sheath. She underwent treatment with a combination of chemotherapy (Paclitaxel) and anti-HER2 monoclonal antibodies (Trastuzumab).

**Results:**

Nine months after treatment, a B-scan showed resolution of the superior choroidal focus, as well as absence of blood flow within the optic nerve sheath. The inferonasal lesion was still present but the dimensions were reduced.

**Conclusion:**

The patient underwent a combined treatment of chemotherapy and Trastuzumab to increase the response rate. Trastuzumab is a humanized monoclonal antibody, which binds to the extracellular segment of the HER2/neu receptor. Nine months following the therapy her vision was stable, whilst one focus of the tumour in the affected eye, had regressed. The favourable response highlights the significant impact of this new therapy, as an alternative to external beam radiotherapy in patients with ocular metastasis from HER2 (+) breast cancer.

## Introduction

Breast cancer is an increasing important health problem in women, and is the most common tumour to metastasize to the uveal tract, presumably due to its large blood supply. Unfortunately, the mean survival period after diagnosis of ocular metastasis, ranges from 10 to 32 months [1,2]. However, recent advances in therapy including monoclonal antibodies (Trastuzumab) with targeted effects, are likely to improve treatment outcomes and prolong survival rates.

## Case Report

A 45 year old female with a history of metastatic breast cancer diagnosed in 1995, and treated with mastectomy and chemotherapy, was referred for an ophthalmological opinion. She had recently developed deterioration of vision in the left eye. The patient, 3 months prior to referral, had undergone treatment with Trastuzumab and Taxotere, as well as brain radiotherapy (20 Gy in 5 fractions) for a solitary metastasis on the right occipital lobe. A series of subsequent scans had shown almost complete response.

**Figure 1:**
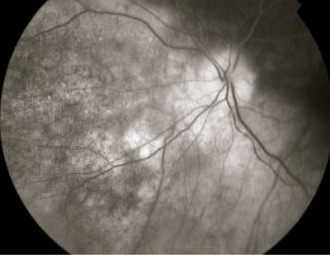
**Fluorescein angiography of the left eye showing early hyperflourescence with central masking inferonasal to the optic disc**.

**Figure 2:**
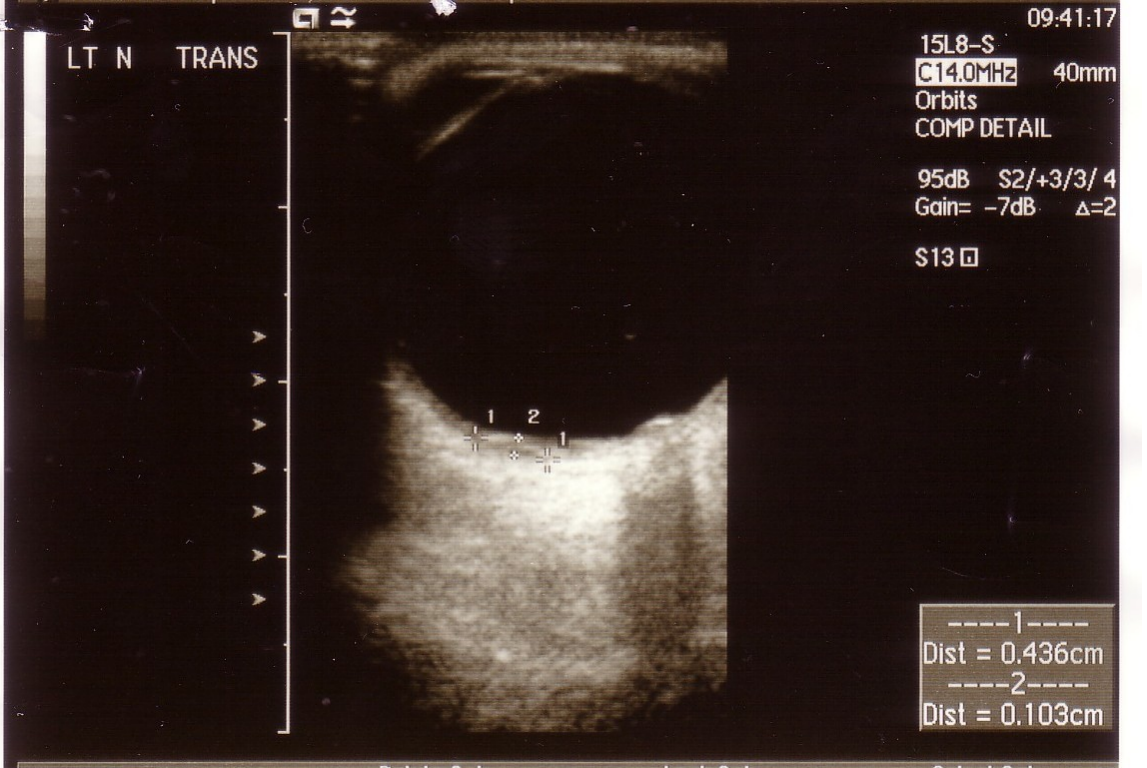
**B-scan of the left eye before treatment showing the superior lesion (4.4 mm transverse base, 3.9 mm longitudinal base and 1.0 mm elevation)**.

At presentation, the visual acuity on the left eye was 6/9 with head posture and there was a left RAPD. Fundoscopy showed a white choroidal lesion with central hyper pigmentation, inferonasal and adjacent to the left optic nerve. In addition, there was optic disc pallor. A visual field test showed a left superior altitudinal defect. Fluorescein angiography showed early hyperflourescence with central masking that was highly suggestive of metastatic involvement of the choroid (Fig 1). A B-scan was performed, showing two elevated lesions superior (Fig 2) and inferonasal (3.3 mm transverse, 2.8 mm longitudinal base and 1.0 mm elevation) to the optic disc. In addition blood flow was detected in the optic nerve sheath, suggesting tumour extension to the optic nerve.

**Figure 3:**
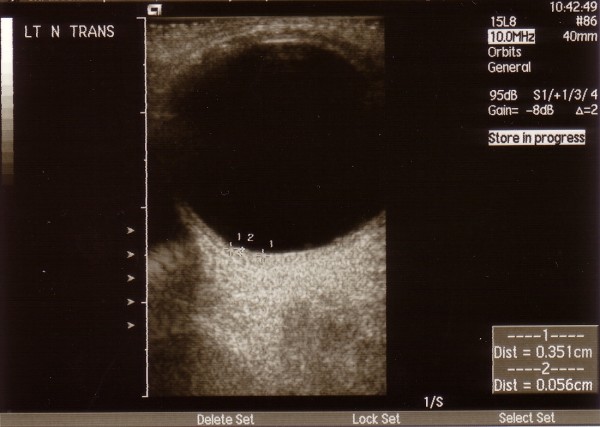
**B-scan 8 months post-treatment showing the reduced dimensions of the inferonasal lesion (3.5 mm transverse, 2.3 mm longitudinal base and 0.6 mm elevation)**.

The patient was commenced on Paclitaxel and Trastuzumab at four weekly intervals, and was responding well with very little systemic toxicity. Nine months later her visual acuity on the left eye was 6/9 without the head posture with persistence of the left visual field defect. A subsequent B-scan showed complete resolution of the superior focus, as well as absence of blood flow within the optic nerve sheath. The inferonasal lesion (Fig 3) was still present but the dimensions were reduced (3.5 mm transverse, 2.3 mm longitudinal base and 0.6 mm elevation). Enhanced scans were performed and showed absence of metastatic disease, whilst a bone scan showed significant reduction of isotope uptake. The patient is currently on Trastuzumab three weekly and under combined follow up.

## Discussion

The incidence of ocular metastasis from breast cancer ranges from 9% to 37%. It also accounts for 39% to 49% of all uveal metastases. There are few reports about the response of choroidal metastases to chemotherapy and radiotherapy. A recent study at the Oncology service of Wills Eye Hospital reported that the average survival time after the diagnosis of ocular metastasis was 65% at 1 year and 24% at 5 years. However, survival rates depend on general health, early diagnosis and advances in therapy [2,3].

Trastuzumab, is a humanized monoclonal antibody which binds to the extracellular segment of the HER2/neu (erbB2) receptor [4]. Despite the controversy for public health funding in UK, it was approved by the NICE in June 2006 (following its European licence) and its initiation is based upon identification of HER-2 over expression. The combination of Trastuzumab with chemotherapy has been shown to increase both survival and response rate, in comparison to Trastuzumab alone. Recent trials have shown 50% reduction of the relapse risk in the adjuvant setting (after the initial diagnosis) for one year [5].

In our patient the ocular lesion was diagnosed relatively early, and she underwent a combined treatment of chemotherapy and Trastuzumab to increase the response rate. Nine months following therapy her condition was stable and her vision was preserved.

## Conclusion

Though further follow up is essential, this case report highlights the significant impact of monoclonal antibody therapy, as an alternative to external beam radiotherapy in patients with ocular metastasis from HER2 (+) breast cancer.

## Abbreviations

HER2/neu: Human Epidermal Growth Factor Receptor 2; RAPD: Relative Afferent Papillary Defect; NICE: National Institute for Clinical Excellence.

## Consent

Written informed consent was obtained from the patient for publication of this case report and accompanying images. A copy of the written consent is available for review by the Editor-in-Chief of this journal

## Competing interests

The authors declare that they have no competing interests.

## Authors' contributions

KP and AI reviewed the case and wrote the paper, AS and ND were directly involved with the patient's treatment and were major contributors in the manuscript. All authors read and approved the final manuscript.
